# The case of an 81‐year‐old man with left toes paralysis after cardiac procedure

**DOI:** 10.1002/acn3.51323

**Published:** 2021-03-03

**Authors:** Keng Lam, Manya Khrlobyan, Navdeep Sangha

**Affiliations:** ^1^ Department of Neurology Kaiser Permanente, Los Angeles Los Angeles California USA; ^2^ Department of Neurology University of Southern California, Los Angeles Los Angeles California USA

## Summary of Case

An 81‐year‐old male with a past medical history of diabetes and aortic valve stenosis status posttranscatheter aortic valve implantation day 0 reported sudden inability to move his left toes several hours after the procedure. The nursing staff immediately notified the overnight neurology resident for an urgent evaluation. His neurological examination was grossly normal including left foot dorsiflexion, plantar flexion, inversion, and eversion, except for the fact that he was not able to flex or extend toes 1–5 on the left side. A CT scan of the abdomen and pelvis was ordered which was unremarkable without evidence of a retroperitoneal hematoma. MRI brain demonstrates bilateral acute ischemic infarcts including the right paracentral lobule localizing to paralysis of the left toes. Our case illustrates the importance of having a high index of suspicion for an ischemic stroke in a patient who is in the postoperative setting, especially if the procedure is cardiac related.

**Figure 1:**
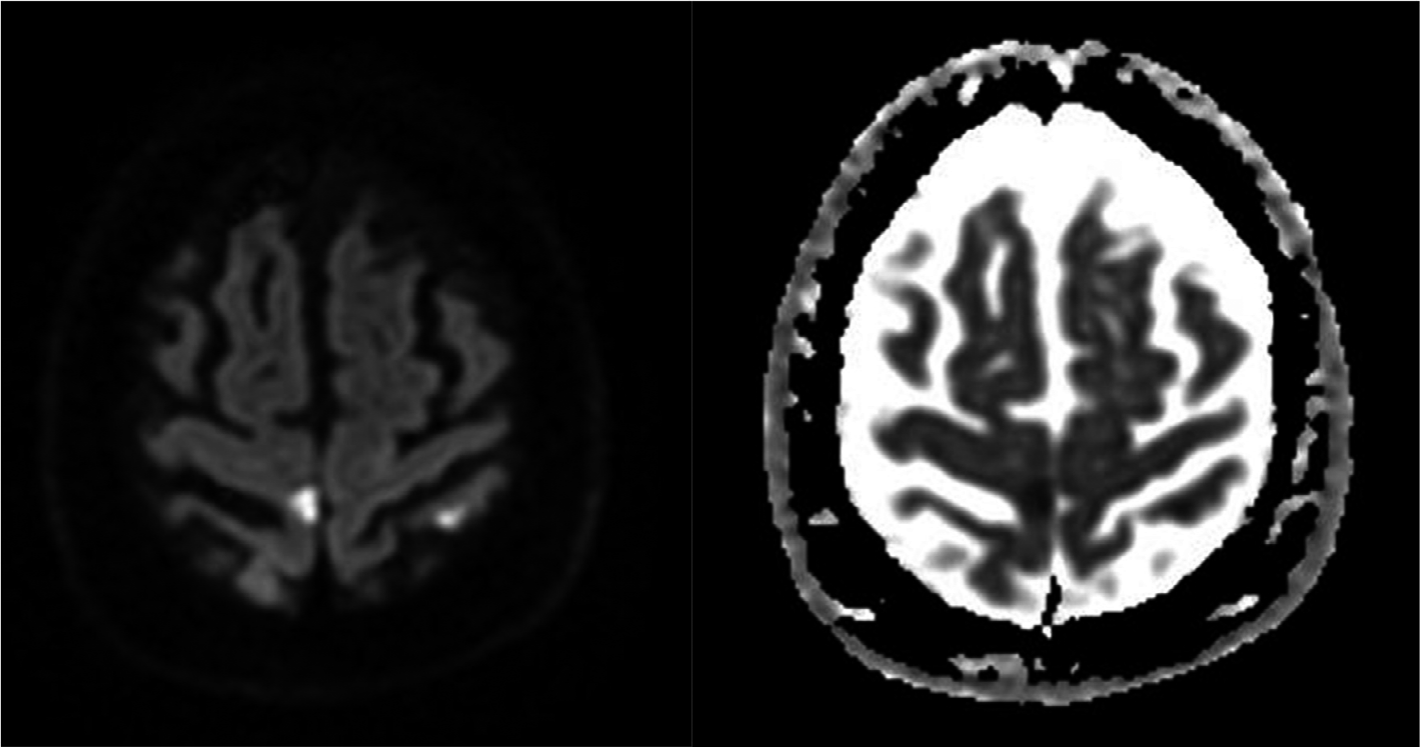
DWI bright/ADC dark signal suggests an acute infarct in the right paracentral lobule, likely embolic, which corresponds to the left toe weakness.

*Diagnosis:* Paracentral lobule infarct of cardioembolic etiology associated with postcardiac procedure.

## Take‐Home Points

- Lower extremity weakness after catheterization procedure should prompt consideration of a retroperitoneal hematoma.
- Cardiac procedure, such as transcatheter aortic valve implantation, carries a risk for ischemic stroke.^1^
- Isolated monoparesis of the toes due to embolic stroke has been reported and can be caused by a lesion in the contralateral paracentral lobule.^2^, ^3^
- Despite a complete neurological exam, localization for an ischemic stroke can be difficult and it is important to place substantial consideration on the patient's risk factors.
